# Breastfeeding is associated with enhanced intestinal gluconeogenesis in infants

**DOI:** 10.1186/s12916-024-03327-w

**Published:** 2024-03-07

**Authors:** Duan Ni, Jian Tan, Laurence Macia, Ralph Nanan

**Affiliations:** 1 Charles Perkins Centre, The University of Sydney, Sydney, NSW Australia; 2 Sydney Medical School Nepean, Nepean Hospital, The University of Sydney, Level 5, South Block, Penrith, Sydney, NSW 2751 Australia; 3 Nepean Hospital, Nepean Blue Mountains Local Health District, Penrith, NSW Australia; 4 School of Medical Sciences, Faculty of Medicine and Health, The University of Sydney, Sydney, NSW Australia; 5 Sydney Cytometry Core Research Facility, Charles Perkins Centre, The University of Sydney and Centenary Institute, Sydney, NSW Australia

**Keywords:** Breastfeeding, Formula feeding, Metabolic health, Intestinal gluconeogenesis, Infants

## Abstract

**Background:**

Breastfeeding (BF) confers metabolic benefits to infants, including reducing risks of metabolic syndrome such as obesity and diabetes later in life. However, the underlying mechanism is not yet fully understood. Hence, we aim to investigate the impacts of BF on the metabolic organs of infants.

**Methods:**

Previous literatures directly studying the influences of BF on offspring’s metabolic organs in both animal models and humans were comprehensively reviewed. A microarray dataset of intestinal gene expression comparing infants fed on breastmilk versus formula milk was analyzed.

**Results:**

Reanalysis of microarray data showed that BF is associated with enhanced intestinal gluconeogenesis in infants. This resembles observations in other mammalian species showing that BF was also linked to increased gluconeogenesis.

**Conclusions:**

BF is associated with enhanced intestinal gluconeogenesis in infants, which may underpin its metabolic advantages through finetuning metabolic homeostasis. This observation seems to be conserved across species, hinting its biological significance.

**Supplementary Information:**

The online version contains supplementary material available at 10.1186/s12916-024-03327-w.

## Background

We read with great interest the findings from the paper by Bugaeva et al. describing the lack of evidence supporting the potential protective effect of breastfeeding (BF) on the mental health in both mothers and children [1].

In addition to mental health, BF’s impacts towards the infants’ metabolic health are also of great interest. BF delivers several advantages to infants’ metabolism. It programs their metabolic health, reducing the risk of obesity, diabetes, and the metabolic syndrome later in life [2, 3]. However, studies investigating these associations have primarily been observational. A deeper mechanistic understanding of direct effects of BF on metabolic organs at a cellular level are lacking in humans. Here, we harnessed a published microarray dataset comparing intestinal gene expression between BF and formula feeding (FF) infants [4]. Comprehensive profiling their metabolic landscapes revealed enhanced intestinal gluconeogenesis (IGN) in BF infants. Our results align with studies in other mammalian species showing similar effects of BF on gluconeogenesis in metabolic organs. Since IGN, which is induced shortly post birth [5], contributes to modulating energy homeostasis, appetite, and insulin sensitivity [6], our data provides some potential mechanistic insights for the metabolic advantages reported in breastfed infants at a cellular level in humans.

## Methods

For analyses in animal models, gene expression data was extracted from original papers [7–10]. For analysis of the human infant study, processed microarray data was downloaded from [4] (GEO ID: GSE31075) and normalized with DESeq2 (v1.34.0) package [11]. The resulting data was subject to further analyses.

Expression of key genes involved in gluconeogenesis pathway were comparatively analyzed with PRISM GraphPad. Their corresponding false discovery rates (FDRs) were calculated with DESeq2. Gene set score was computed with *gsva* (v1.51.5) package [12] based on the “Human Gene Set: Reactome Gluconeogenesis” gene set data from the Gene Set Enrichment Analysis (GSEA) software [13]. Finally, GSEA was run with the GSEA (v4.3.2.) software. All analyses were carried out according to the appropriate packages/software’s tutorials.

For metagenomic analysis, raw sequences minus human-identical sequences were downloaded from [4] (ERP001038). Sequences were first quality filtered with *fastp* [14] with the default parameters to ensure the removal of low-quality reads, duplicated reads, and adapter sequences, prior to downstream analysis. After that, sequences were clustered using CDHIT-454 and taxonomic assignment on resulting reads were performed using MetaPhlAn2. The HUMAnN2 pipeline was utilized for functional profiling to identify gene families and MetaCyc metabolic pathways.

## Results

**Fig. 1:**
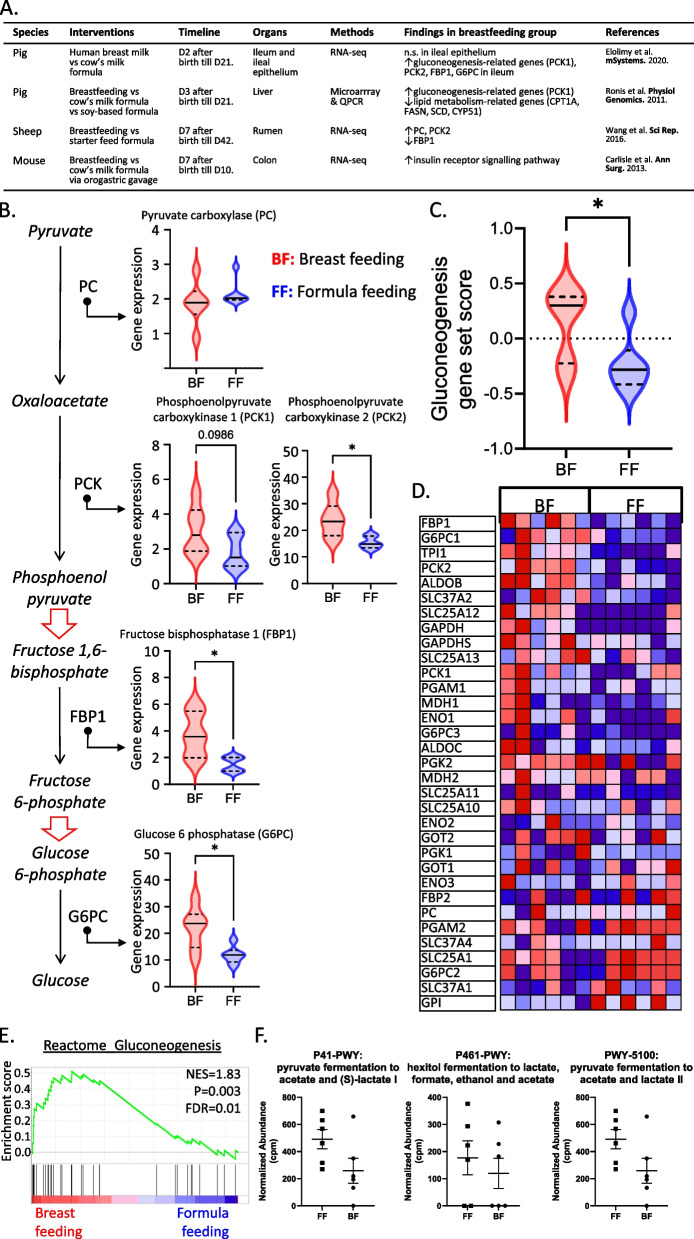
Breast feeding (BF) enhanced gluconeogenesis in infants from both animal models and humans. **A** Overview of animal studies investigating the impacts of BF on the offspring’s metabolisms. **B** Overview of the critical steps of gluconeogenesis pathway and the expression of critical genes involved in BF (red) versus formula feeding (FF, blue) infants’ intestinal cells. Statistical significance was achieved in both unpaired *t*-test and Wilcoxon test. PCK2, false discovery rate (FDR) = 0.12; G6PC, FDR = 0.07. Solid lines in the violin plots denote the median expression level and the dashed lines are for the upper and lower quantiles. **C** Gluconeogenesis gene set scores of BF (red) and FF (blue) samples based on the 35 genes from the “Reactome Gluconeogenesis” gene set. Statistical significance was achieved in both unpaired *t*-test and Wilcoxon test. **D** Heatmap overview of the expression pattern of the genes from the “Reactine Gluconeogenesis” gene set in BF (left) and FF (right) samples. **E** Gene set enrichment analysis (GSEA) showed the enrichment of genes involved in gluconeogenesis pathways in BF (red) infants’ intestinal cells. **F** Scattering plots for the normalized abundance of the corresponding pathways predicted from microbiota analysis comparing BF (round dot) and FF (square dot) infants. No difference was detected (n.s., no significant change; PCK1, phosphoenolpyruvate carboxykinase 1, PCK2, phosphoenolpyruvate carboxykinase 2, FBP1, fructose bisphosphatase 1, G6PC, glucose 6 phosphatase, CPT1A, carnitine palmitoyltransferase 1, FASN, fatty acid synthase, SCD, stearoyl-CoA desaturase, CYP51, cholesterogenic lanosterol 14alpha-demethylase, PC, pyruvate carboxylase, NES, normalized enrichment score, FDR, false discovery rate)

We first collated evidence for cellular metabolism impacted by BF in non-human mammalian species (Fig. 1A). In studies involving pigs, BF increased gluconeogenesis in offspring's ileum and liver, evidenced by the upregulation of key genes in this pathway, including phosphoenolpyruvate carboxykinase 1 (PCK1) [7, 8]. In breast-fed lambs, there was an increase in PCK2 expression in the rumen [9]. Furthermore, the insulin receptor signaling pathway was found to be activated in the colon of breastfed mice [10].

Subsequently, leveraging a published microarray dataset, we comprehensively interrogated the impacts of BF versus FF on the intestinal metabolism of human infants. Six BF and six FF infants, appropriately balanced (Additional File 1: Table S1), were analyzed.

We found that several critical genes involved in gluconeogenesis, such as PCK2, fructose bisphosphatase 1 (FBP1), and glucose 6 phosphatase (G6PC), were increased in BF samples, implying enhanced IGN (Fig. 1B). We also calculated the gluconeogenesis gene set score based on the “Reactome Gluconeogenesis” gene set. As shown in Fig. 1C, the BF group exhibited significantly higher gluconeogenesis gene set scores, again indicating that BF was associated with higher IGN. We further carried out GSEA analysis. This unveiled that gluconeogenesis signals were enriched in the BF group (Fig. 1D–E). Together, these data showed that compared with FF, BF was linked to increased IGN in infants.

One of the main triggers of IGN are the gut microbial metabolites, like short-chain-fatty-acids (SCFAs). Harnessing the metagenomic data from the same study, we probed into the microbial metabolic pathways implicated in SCFA production. As shown in Fig. 1F, no difference was found for these pathways between BF and FF samples.

## Discussions

The intestine is an important metabolic organ, responsible for nutrient digestion and absorption. It is also a critical site for gluconeogenesis in addition to liver and kidney, maintaining metabolic homeostasis [6]. Here, we re-analyzed the intestinal microarray data from BF and FF infants. Harnessing the state-of-the-art analytical tools like GSEA and gene set score analysis, which facilitate more in-depth and comprehensive appreciations of the transcriptomic data, we uncovered that BF was associated with enhanced IGN, possibly contributing to improved metabolic health. Importantly, the BF-related enhanced IGN seems to be conserved across species, hinting its biological significance.

Previous studies analyzing this dataset uncovered that UCP2, a critical protein involved in glucose and energetic metabolism [15], was altered comparing BF with FF samples [16]. This partly supported our results that BF was associated with changes in intestinal glucose metabolism.

IGN is essential for maintaining overall glucose balance. It specifically decreases lipid accumulation in the liver, averting liver steatosis, and promotes thermogenesis in adipose tissues and the conversion of white fat to brown fat [6]. Additionally, glucose from IGN, when detected by the portal vein, can modulate food intake [6]. These processes may in part explain the metabolic advantages seen in BF infants, such as reduced risks of metabolic disorders later in life.

The exact mechanisms by which BF might stimulate IGN remain elusive. While dietary protein is a known stimulant for IGN [6], the slightly lower protein content in breast milk compared to infant formula suggests other factors to be at play. Alternatively, metabolites from the infants’ gut bacteria, like acetate and succinate, serve as important IGN fuels [17]. However, our microbiota analysis indicated so far only insignificant differences in their production pathways between BF and FF infants. Further confirmations with methods like metabolomics are warranted. Conversely, breast milk itself is rich in maternal microbial metabolites [18], which may explain the described changes.

Collectively, we provide robust evidence for enhanced IGN possibly triggered by BF in humans on a small sample size. However, our results shed further light on the benefits of BF and justify future research to unravel the breast milk components responsible for IGN induction and to determine how long the increased IGN persists over time.

## Conclusions

BF is associated with enhanced IGN in infants. This might underpin the long-term metabolic benefits conferred by BF. It warrants more in-depth research to elucidate the detailed mechanisms, which might further support the existing recommendations to encourage breastfeeding.

### Supplementary Information

**Additional file 1: Table S1.** Infant growth characteristics.

## Data Availability

RNA-seq data is available as Gene Expression Omnibus ID: GSE31075, and metagenomic data is available as European Bioinformatics Institute’s Short Read Archive study accession number: ERP001038.

## Abbreviations

BF: Breastfeeding
FF: Formula feeding
IGN: Intestinal gluconeogenesis
PCK1: Phosphoenolpyruvate carboxykinase 1
PCK2: Phosphoenolpyruvate carboxykinase 2
FBP1: Fructose bisphosphatase 1
G6PC: Glucose 6 phosphatase
CPT1A: Carnitine palmitoyltransferase 1
FASN: Fatty acid synthase
SCD: Stearoyl-CoA desaturase
CYP51: Cholesterogenic lanosterol 14alpha-demethylase
PC: Pyruvate carboxylase
NES: Normalized enrichment score
FDR: False discovery rate
SCFAs: Short-chain-fatty-acids
