# Supplementary material for: Breastfeeding is associated with enhanced intestinal gluconeogenesis in infants
Source: BMC Med. 2024 Mar 7;22:106. doi: 10.1186/s12916-024-03327-w (PMC10921696; doi:10.1186/s12916-024-03327-w)
Supplement: Supplementary file 1 — Additional file 1: Table S1. Infant growth characteristics. [file 12916_2024_3327_MOESM1_ESM.pdf]

Additional File

Additional file 1: Table S1. Infant growth characteristics.

	Breast feeding	Formula feeding
**Maternal age**	30.0±4.6	30.7±5.9
**Parity**	2.0±0.00	2.1±0.6
**Body weight (kg)**		
**At birth**	3.79±0.50	3.46±0.20
**Month 1**	4.98±0.73	4.61±0.65
**Month 2**	6.43±0.73	5.66±0.85
**Month 3**	7.02±0.72	6.45±0.96
**Diet intake (ml/kg/d)**		
**Month 1**	166.0±18.3	162.5±28.4
**Month 2**	127.6±19.5	138.5±14.3
**Month 3**	129.0±20.1	134.8±9.5
**Growth rate**		
**Month 1**	31.4±3.9%	33.2±2.3%
**Month 2**	69.7±3.0%	63.4±2.6%
**Month 3**	85.2±2.8%	86.4±2.5%
